# Melanocortin-4 receptor in macrophages attenuated angiotensin II-induced abdominal aortic aneurysm in mice

**DOI:** 10.1038/s41598-023-46831-4

**Published:** 2023-11-13

**Authors:** Kentaro Mori, Hideyuki Okuma, Suguru Nakamura, Hiroyuki Uchinuma, Shigeaki Kaga, Hiroyuki Nakajima, Yoshihiro Ogawa, Kyoichiro Tsuchiya

**Affiliations:** 1 Interdisciplinary Graduate School of Medicine and Engineering, University of Yamanashi, 1110 Shimokato, Chuo, Yamanashi 4093898 Japan; 2 Department of Surgery II, Faculty of Medicine, University of Yamanashi, Yamanashi, Japan; 3 Department of Medicine and Bioregulatory Science, Graduate School of Medical Sciences, Kyushu University, Fukuoka, Japan

**Keywords:** Cardiology, Endocrinology

## Abstract

Obesity is recognized as an independent risk factor for abdominal aortic aneurysm (AAA). While mutations in the melanocortin-4 receptor (*MC4R*) gene is the most common cause of obesity caused by mutations in a single gene, the link between MC4R function and vascular disease has still remained unclear. Here, by using melanocortin-4 receptor (MC4R) deficient mice, we confirmed MC4R deficiency promotes AAA and atherosclerosis. We demonstrated the contribution of two novel factors towards vascular vulnerability in this model: leptin signaling in vascular smooth muscle cells (VSMCs) and loss of MC4R signaling in macrophages. Leptin was shown to promote vascular vulnerability via PI3K-dependent upregulation of *Spp1* expression in VSMC. Additionally, Ang II-induced AAA incidence was significantly reduced when *MC4R* gene expression was myeloid cell-specifically rescued in MC4R deficient (MC4R^*TB/TB*^) mice. Ex vivo analysis showed a suppression in NF-κB activity in bone marrow-derived macrophages from LysM(+);MC4R^*TB/TB*^ mice compared to LysM(−);MC4R^*TB/TB*^ mice, which exaggerates with endogenous MC4R ligand treatment; α-MSH. These results suggest that MC4R signaling in macrophages attenuates AAA by inhibiting NF-κB activity and subsequent vascular inflammation.

## Introduction

Extensive research conducted over the last few decades has demonstrated that obesity is a common underlying pathophysiological condition in several diseases, including type 2 diabetes, atherosclerotic disease, chronic kidney disease, and various cancers. More than 650 million adults worldwide currently suffer from obesity (body mass index [BMI] ≥ 30 kg/m^2^), a figure that has tripled over the past four decades. Additionally, several studies have revealed that obesity is an independent risk factor for abdominal aortic aneurysm (AAA)^1, 2^. The presence of AAA or an increasing abdominal aortic diameter was observed to be positively associated with the anthropometric measurements of BMI and waist circumference^1^. Additional research using multivariable analysis demonstrated that in individuals with an increased waist circumference had a 30% higher risk for developing AAA than people with a normal waist circumference, indicating that abdominal adiposity is associated with an increased risk for AAA^3^. More interestingly, a recent systematic analysis of human observational studies demonstrated that people diagnosed with AAA have significantly higher levels of perivascular adipose tissue (PVAT) to abdominal adipose tissue ratio and the circulating leptin concentration than controls^2^. Locally applied leptin in the periaortic area of the mice was shown to augment medial matrix metallopeptidase (MMP)-9 synthesis and aortic aneurysm size^4^, suggesting a key role of leptin in promoting AAA development associated with obesity. However, the precise molecular mechanism linking obesity to AAA has not yet been revealed.

Obesity in a small fraction of cases is known to be caused by mutations in a single gene, resulting in a severe early-onset obesity. The most common cause is mutations in the melanocortin-4 receptor (*MC4R*) gene and up to 5% of patients with severe obesity during childhood are known to carry the pathogenic mutations causing *Mc4r* deficiency^5–7^. Subjects with *MC4R* deficiency exhibit early-onset hyperphagia^7^. In addition to increased fat mass, they exhibit more lean mass and higher bone mineral density; moreover, they are taller than non-*Mc4r*-deficient obese subjects^8^. The severity of these clinical phenotype varies depending upon the functional implications of receptor mutation^5^. In a genetic association study conducted by a British group on the data of approximately 0.5 million people available from UK Biobank that focused on 61 nonsynonymous variants found in *MC4R,* they identified a subset of individuals as carriers for gain-of-function alleles exhibiting lower BMI and obesity, type 2 diabetes, and coronary artery disease^9^. While it is still debating whether genetic variation in MC4R affects cardiovascular disease outcomes in human^10, 11^, MC4R loss of function is found to be related to vascular diseases in mice. This study demonstrated that in comparison to LDL receptor-deficient mice, *Mc4r*- and the LDL receptor-double-deficient mice showed higher developed atherosclerosis^12^. Although these previous reports suggest the link between MC4R function and vascular disease, it seems the evidences are insufficient yet. Therefore, the primary aim of this study is to investigate whether loss of MC4R exacerbates vascular diseases, mainly focusing on AAA by using *Mc4r*-deficient mice. If this is the case, we further aim to examine the molecular mechanism by which MC4R signaling is involved in the development of vascular diseases.

## Results

### MC4R deficiency leads to vascular vulnerability, and promotes Ang II-induced AAA in mice

**Figure 1:**
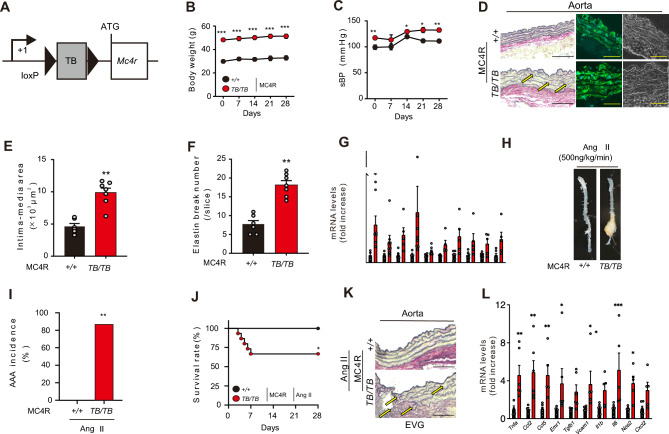
WD-fed MC4R^*TB/TB*^ mice develop vascular vulnerability and Ang II-induced AAA. (**A**) Schematic of MC4R transcription blocking (TB) casette in MC4R^*TB/TB*^ mice. The *Mc4r* coding region is located after TB cassette flanked by loxP sites (represented by triangles). (**B**) Bodyweight and (**C**) systolic blood pressure (sBP) of 14–18-weeks-old WD-fed MC4R^+*/*+^ and MC4R^*TB/TB*^ mice (n = 6–7). (**D**) Representative fluorescent immunostaining images of Elastica van Gieson (EVG) (left), α-smooth muscle actin (middle), and phase difference images (right) in the aorta of 18-weeks-old WD-fed MC4R^+*/*+^ and MC4R^*TB/TB*^ mice (black scale bars represent 100 μm, yellow scale bars represent 50 μm). Quantification of (**E**) the intima–media area and (**F**) elastin break number (n = 6–7) in the aorta. (**G**) Gene expression in aorta of WD-fed MC4R^+*/*+^ and MC4R^*TB/TB*^ mice (scale bars represent 20 μm). (**H**) Representative pictures of the aorta and (**I**) AAA incidence of Ang II (500 ng/kg/min, from 14- to 18-weeks-old) -infused WD-fed MC4R^+*/*+^ and MC4R^*TB/TB*^ mice. (n = 11–15). (**J**) Kaplan–Meier curve of the survival rate in Ang II-infused WD-fed MC4R^+*/*+^ and MC4R^*TB/TB*^ mice. (**K**) Representative pictures of EVG in the aorta (n = 11–15, scale bars represent 100 μm). (**L**) Gene expression in aorta of Ang II-infused WD-fed MC4R^+*/*+^ and MC4R^*TB/TB*^ mice. *P < 0.05, **P < 0.01, and ***P < 0.001.

To create MC4R-defecient mice, we used a model wherein loxP-flanked transcriptional blocking (TB) cassette is inserted upstream of the *Mc4r* ATG site, resulting in the disruption of *Mc4r* expression (Fig. 1A)^13, 14^. Western diet (WD)-fed MC4R^*TB/TB*^ mice exhibited significantly higher bodyweight (Fig. 1B) and sBP (Fig. 1C) in comparison to WD-fed MC4R^+/+^ mice. While WD-fed MC4R^*TB/TB*^ mice did not show any presence of AAA, histochemistry analysis revealed an increase in the intima–media area and elastin break number in the aorta of them, suggesting vascular vulnerability (Fig. 1D–F). Moreover, several inflammatory-related genes were upregulated in the aorta of WD-fed MC4R^*TB/TB*^ mice (Fig. 1G).

Further, to determine whether these findings in MC4R^*TB/TB*^ mice contribute towards AAA, we first treated WD-fed MC4R^*TB/TB*^ and MC4R^+*/*+^ mice with Ang II (1000 ng/kg/min—usual dose to induce AAA in mice) for 4 weeks^15, 16^. However, this dose resulted in a mortality rate of more than 80% in WD-fed MC4R^*TB/TB*^, primarily due to the AAA rupture within the first 2 weeks (data not shown), suggesting that WD-fed MC4R^*TB/TB*^ mice were markedly susceptible for AAA. Therefore, to induce AAA, we chose 500 ng/kg/min of Ang II instead of 1000 ng/kg/min.

While higher bodyweight and sBP were observed in MC4R^*TB/TB*^ compared to the MC4R^+*/*+^ mice (Supplementary Fig. 1A,B), higher elastin break numbers and AAA incidence (Supplementary Fig. 1C,H,I,K) along with a lower survival rate (Fig. 1J) were found in MC4R^*TB/TB*^ mice in comparison to MC4R^+*/*+^ mice fed a WD. An increased AAA incidence and diameter were observed in MC4R^*TB/TB*^ mice compared to MC4R^+*/*+^ mice, even after matching of the sBP levels in MC4R^*TB/TB*^ and MC4R^+*/*+^ mice using hydralazine (Supplementary Fig. 1D–F). Additionally, compared to MC4R^+*/*+^ mice, MC4R^*TB/TB*^ mice showed increased inflammatory-related gene expression (Fig. 1L), as well as the positive F4/80 immunostaining area (Supplementary Fig. 1G,H) in the aorta.

### MC4R deficiency promotes atherosclerosis and atherosclerosis-associated AAA in apolipoprotein E-knockout mice

**Figure 2:**
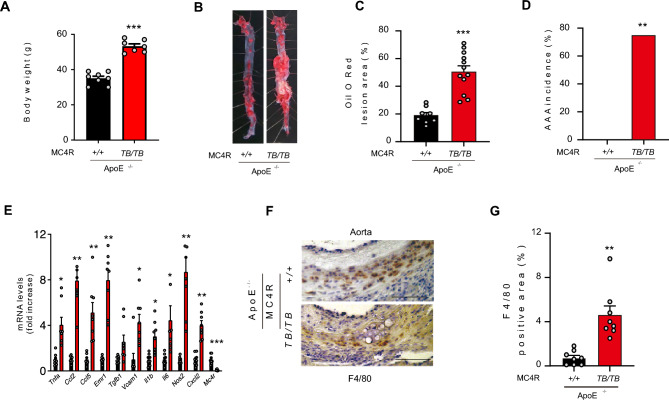
MC4R^*TB/TB*^ mice in an ApoE^−/−^ background develop atherosclerosis. (**A**) Bodyweight of SD-fed MC4R^+*/*+^ and MC4R^*TB/TB*^ 18-weeks-old mice in an ApoE^*-*^background (n = 7–8). (**B**) Representative pictures of the aorta with Oil O Red staining. (**C**) Quantification of the lesion area in 18-weeks-old SD-fed ApoE^*−/−*^;MC4R^+*/*+^ and ApoE^*−/−*^;MC4R^*TB/TB*^ mice (n = 8–12). (**D**) AAA incidence. (**E**) Gene expression in the aorta of the SD-fed ApoE^*−/−*^;MC4R^+*/*+^ and ApoE^*−/−*^;MC4R^*TB/TB*^ mice (n = 8). (**F**) Representative pictures and (**G**) quantification of the F4/80 immunostaining in the aorta (n = 8). *P < 0.05, **P < 0.01, ***P < 0.001.

To explore accessorily whether MC4R deficiency promotes atherosclerosis, we crossed MC4R^*TB/TB*^ mice with apolipoprotein E (ApoE) knockout (ApoE^*−/−*^) mice to generate double-deficient MC4R and ApoE (ApoE^*−/−*^;MC4R^*TB/TB*^) mice. ApoE^*−/−*^;MC4R^*TB/TB*^ mice fed a standard chow exhibited higher bodyweight (Fig. 2A) and concentrations of total cholesterol, triglyceride (TG), blood glucose, and serum insulin than control ApoE^*−/−*^;MC4R^+*/*+^ mice (Supplementary Fig. 2A–E). In comparison to ApoE^*−/−*^;MC4R^+*/*+^ mice, ApoE^*−/−*^;MC4R^*TB/TB*^ mice exhibited exaggerated atherosclerosis (Fig. 2B,C). Surprisingly, ApoE^*−/−*^;MC4R^*TB/TB*^ mice also showed a higher incidence of AAA associated with atherosclerosis (Fig. 2D), increased proinflammatory genes (Fig. 2E), and immune-positive areas for F4/80 (Fig. 2F,G) without Ang II administration.

### MC4R deficiency promotes Ang II-induced AAA formation via leptin-dependent and -independent mechanisms

**Figure 3:**
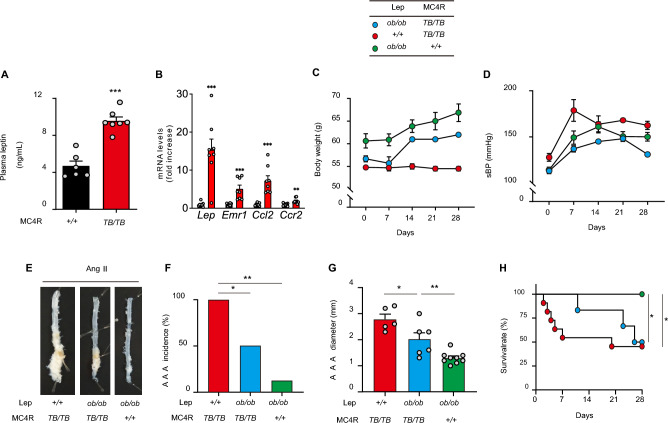
MC4R deficiency promotes Ang II-induced AAA formation via leptin-dependent and -independent mechanisms. (**A**) Plasma concentration of leptin in 14-weeks-old, WD-fed MC4R^+*/*+^ and MC4R^*TB/TB*^ mice (n = 6–7). (**B**) Gene expression in the abdominal perivascular adipose tissue in 14-weeks-old WD-fed MC4R^+*/*+^ and MC4R^*TB/TB*^ mice (n = 7–8). Changes in (**C**) bodyweight and (**D**) systolic blood pressure (sBP) in WD-fed MC4R^*TB/TB*^, *ob/ob*, and *ob/ob*;MC4R^*TB/TB*^ mice during Ang II (500 ng/kg/min) infusion from 14 to 18 weeks of age (n = 5–8 at the beginning of Ang II infusion). (**E**) Representative pictures of the aorta and (**F**) AAA incidence and (**G**) diameter after Ang II infusion (n = 5–9). (**H**) Kaplan–Meier curve of the survival rate in WD-fed MC4R^*TB/TB*^, *ob/ob*, and *ob/ob*;MC4R^*TB/TB*^ mice during Ang II (500 ng/kg/min) infusion. *P < 0.05, **P < 0.01, ***P < 0.001.

Next, to dissect the molecular mechanism by which MC4R signaling is involved in the development of vascular diseases, we determined to focus on Ang II-induced AAA model because this model is considered to take shorter time period to conduct than atherosclerosis model with the background of ApoE^*−/−*^;MC4R^*TB/TB*^. In the arcuate nucleus of the hypothalamus, MC4R plays a pivotal role in mediating appetite suppression via the effect of leptin^17^:Consistently, MC4R deficiency resulted in hyperleptinemia in the peripheral blood, possibly via negative feedback (Fig. 3A). We also confirmed that leptin and several proinflammatory genes are significantly upregulated in abdominal perivascular adipose tissue (PVAT) of WD-fed MC4R^*TB/TB*^ mice compared to that of WD-fed MC4R^+*/*+^ mice (Fig. 3B). Since leptin has been reported to stimulate vascular inflammation, oxidative stress, and vascular smooth muscle hypertrophy that may contribute to hypertension, vascular injury, and AAA^18^, we investigated whether leptin contributes to vascular vulnerability as observed in MC4R^*TB/TB*^ and ApoE^*−/−*^;MC4R^*TB/TB*^ mice by comparing the three groups of mice including leptin-deficient *ob/ob* mouse; *ob/ob*;MC4R^*TB/TB*^, MC4R^*TB/TB*^, and *ob/ob* mice.

Among the three mice fed a standard diet^19^, MC4R^*TB/TB*^ mice exhibited a slight decrease in bodyweight (Fig. 3C) and higher sBP (Fig. 3D). Moreover, MC4R^*TB/TB*^ mice showed the highest triglyceride levels (Supplementary Fig. 3B) as well as the highest glucose and serum insulin levels (Supplementary Fig. 3C,D). After treatment with Ang II (500 ng/kg/min), a significant decline in the incidence and size of AAA (Fig. 3E–G), and an increase in the survival rate of *ob/ob*;MC4R^*TB/TB*^ mice were observed compared to MC4R^*TB/TB*^ (Fig. 3E–H). These in vivo findings indicate towards the contribution of leptin in promoting Ang II-induced AAA. In contrast, compared to these in *ob/ob* mice, AAA incidence and size were found to be higher in *ob/ob*;MC4R^*TB/TB*^, indicating towards a possibility of MC4R inhibiting Ang II-induced AAA in leptin-independent manner. To confirm leptin-dependent and -independent contributions, we determined to do following experiments.

### The vascular vulnerability of MC4R^TB/TB^ mice is mediated by an osteopontin receptor CD44

First, we elucidated a leptin-dependent mechanism by which MC4R deficiency promotes vascular vulnerability. In non-alcoholic steatohepatitis (NASH) fibrosis, it has been reported that leptin promotes fibrosis via upregulation of an extracellular matrix glycoprotein, osteopontin (OPN) in hepatic stellate cells (HSCs)^20^. Leptin is also known to upregulate OPN gene (*Spp1*) expression in vascular smooth muscle cells (VSMCs)^21^. Additionally, OPN promotes Ang II-accelerated atherosclerosis and aneurysm in mice^22^.

**Figure 4:**
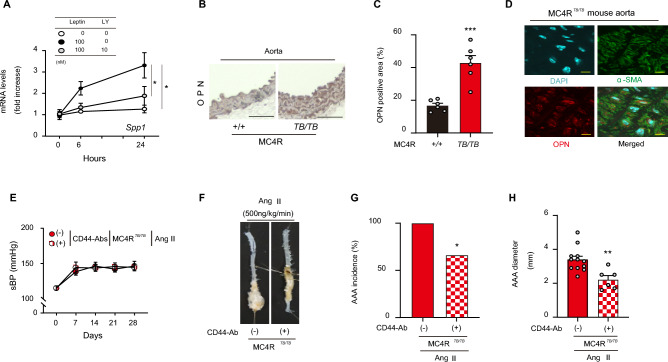
An osteopontin receptor CD44 mediates vascular vulnerability of MC4R^*TB/TB*^ mice. (**A**) Leptin (100 nM)-induced *Spp1* gene expression in primary vascular smooth muscle cells from WT male mice pretreated with or without the PI3K inhibitor LY294002 (LY, 10 nM; n = 4). (**B**) Representative pictures and (**C**) quantification of osteopontin (OPN) immunostaining in the aorta of WD-fed MC4R^+*/*+^ and MC4R^*TB/TB*^ mice (n = 6, scale bar is 100 μm). (**D**) Representative pictures of DAPI, α-smooth muscle cells, and merged images in the aorta of WD-fed MC4R^*TB/TB*^ mice (scale bars represent 20 μm). (**E**) Changes in the sBP of 14–18-weeks-old MC4R^*TB/TB*^ mice during Ang II (500 ng/kg/min) infusion concomitantly administered with anti-CD44 or control antibodies (Ab; n = 6–12). (**F**) Representative pictures of the aorta and (**G**) AAA incidence and (**H**) diameter after Ang II infusion into WD-fed MC4R^+*/*+^ and MC4R^*TB/TB*^ mice administered with anti-CD44 or control Abs (n = 6–12). *P < 0.05, **P < 0.01, ***P < 0.001.

In primary culture of VSMCs from WT mice, leptin upregulated *Spp1* expression, with its effect abolishing by the phosphoinositide 3-kinase inhibitor LY294002 (Fig. 4A) as previously confirmed in HSCs^20^. Immunostaining of the aorta revealed the stronger OPN expression in the vascular walls of MC4R^*TB/TB*^ mice than observed for the MC4R^+*/*+^ mice (Fig. 4B,C). Moreover, OPN-expressing cells colocalized with α-smooth muscle actin-expressing cells (Fig. 4D). Consistent with the observation, plasma leptin concentration was significantly higher in MC4R^*TB/TB*^ mice than in MC4R^+*/*+^ mice and correlated with plasma OPN concentration in MC4R^+*/*+^ and MC4R^*TB/TB*^ mice (Supplementary Fig. 4A,B).

Further, To inhibit OPN action in vivo, we intraperitoneally administered neutralizing antibody against CD44, a cell-surface adhesion receptor of OPN^23^, into Ang II-infused MC4R^*TB/TB*^ mice. The neutralizing antibody significantly inhibited the Ang II-induced AAA in MC4R^*TB/TB*^ mice independent of the sBP (Fig. 4E–H).

### *MC4R* gene reconstitution in myeloid cells suppresses Ang II-induced AAA in MC4R^*TB/TB*^ mice

MC4R is reported to predominantly express in the brain and plays an important role in the regulation of appetite in humans and rodents^6, 17^. However, MC4R is recently reported to express in enteroendocrine L cells and regulate peptide YY and glucagon-like peptide 1 release in mice^24, 25^*.* This finding points toward a notion that MC4R expressed in peripheral cells may directly plays an inhibitory role during AAA development.

**Figure 5:**
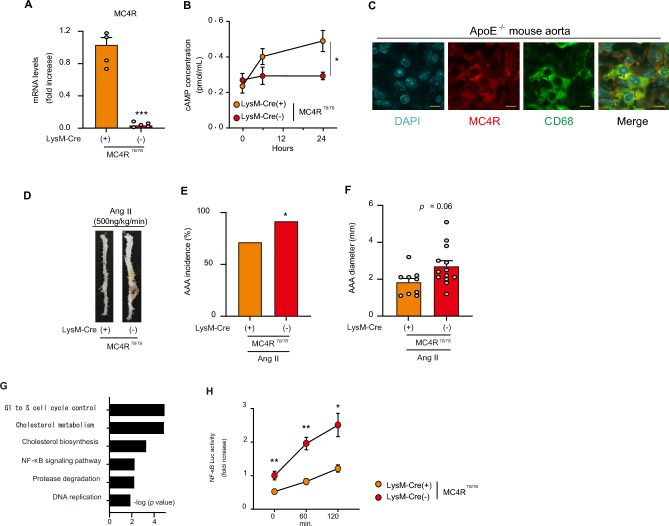
*MC4R* gene reconstitution in myeloid cells suppresses Ang II-induced AAA in MC4R^*TB/TB*^ mice. (**A**) *MC4R* gene expression in terms of mRNA levels in BM-derived macrophages from LysM-Cre (+);MC4R^*TB/TB*^ and LysM-Cre (−);MC4R^*TB/TB*^ mice (n = 4–5). (**B**) Intracellular cAMP concentration in BM-derived macrophages from LysM-Cre (+);MC4R^*TB/TB*^ and LysM-Cre (−);MC4R^*TB/TB*^ treated with α-MSH (1 μM) for different times as indicated in x-axis (n = 4). (**C**) Representative fluorescent immunostainings of Ang II-induced AAA lesions in ApoE^*−/−*^ mice with DAPI, CD68, MC4R, and a merged image (scale bars represent 10 μm). (**D**) Representative pictures of the aorta and (**E**) AAA incidence and (**F**) diameter after Ang II (500 ng/kg/min) infusion at the age of 14–18 weeks (n = 9–13). (**G**) The pathways enriched among the upregulated (> 1.5-fold) mRNAs in BM-derived macrophages from 8-weeks-old LysM-Cre (−);MC4R^*TB/TB*^ mice in comparison to LysM-Cre (+);MC4R^*TB/TB*^ mice. The results are expressed as − log (P value). (**H**) NF-κB luciferase activity in BM-derived macrophages from LysM-Cre (+);MC4R^*TB/TB*^ and LysM-Cre (−);MC4R^*TB/TB*^ mice transduced with adenovirus encoding Nf-κB-responsive luciferase reporter construct followed by LPS (10 ng/mL) stimulation for different times as indicated in x-axis (n = 4). *P < 0.05, **P < 0.01, ***P < 0.001.

Western blotting of various tissues in WT mice indicated that MC4R was expressed in the RAW 264.7 macrophage cell line and bone marrow (BM)-derived macrophages, as well as in the brain, liver, and small intestine (Supplementary Fig. 5A). Therefore, to examine the role of MC4R in macrophages, we created myeloid cell-specific *MC4R* gene reconstitution mice by crossing MC4R^*TB/TB*^ and LysozymeM (LysM)-Cre expression mice (Supplementary Fig. 5B). We successfully confirmed the *MC4R* gene expression in BM-derived macrophages from LysM (+);MC4R^*TB/TB*^ mice (Fig. 5A). On stimulation with α-MSH, an internal ligand of MC4R, while a significant increase in the intracellular cyclic AMP levels in BM-derived macrophages from LysM (+);MC4R^*TB/TB*^ mice was observed, it did not happen in BM-derived macrophages from LysM (−);MC4R^*TB/TB*^ mice (Fig. 5B), suggesting a functional expression of MC4R in macrophages. MC4R was colocalized with macrophage marker CD68-expressing cells in the aorta in ApoE^*−/−*^ mice (Fig. 5C). In vivo, LysM (+);MC4R^*TB/TB*^ mice exhibited reduction in Ang II-induced AAA incidence and diameter compared to LysM (−);MC4R^*TB/TB*^ mice (Fig. 5D–F), which was independent of sBP (Supplementary Fig. 5C).

Further, a microarray analysis in BM-derived macrophages from LysM (+);MC4R^*TB/TB*^ and LysM (−);MC4R^*TB/TB*^ mice revealed nuclear factor-κB (NF-κB) signaling as a significantly ranked pathway (Fig. 5G). LPS-induced NF-κB activity was also significantly suppressed in BM macrophages from LysM (+);MC4R^*TB/TB*^ mice (Fig. 5H). These findings indicate that MC4R in macrophages has a significant contribution in suppressing Ang II-induced AAA, probably via the inhibition of NF-κB activity.

### α-MSH treatment suppresses Ang II-induced AAA in ApoE^−/−^ mice, but not in MC4R^TB/TB^ mice

**Figure 6:**
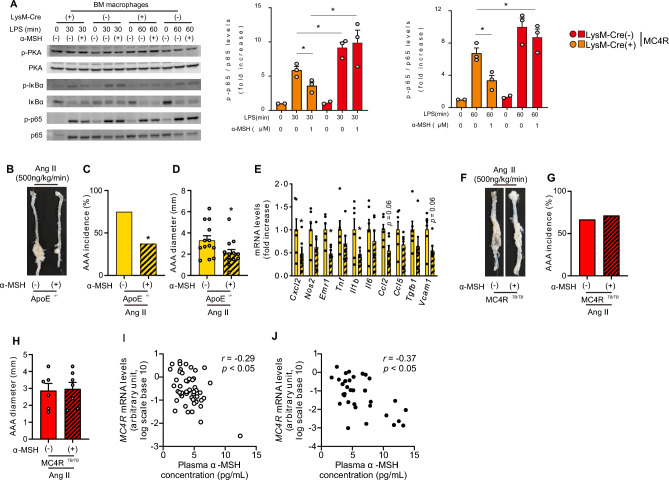
Exogenous α-MSH suppresses Ang II-induced AAA in ApoE^−/−^ mice, but not in MC4R^*TB/TB*^ mice. (**A**) Representative blots and quantification of protein kinase A, IκBα, and p65 phosphorylation in BM-derived macrophages from LysM-Cre (+);MC4R^*TB/TB*^ and LysM-Cre (−);MC4R^*TB/TB*^ mice (n = 2–3). (**B**) Representative pictures of the aorta and (**C**) AAA incidence and (**D**) diameter after Ang II infusion in ApoE^*−/−*^ mice intraperitoneally administered with vehicle and α-MSH (1.0 mg/kg/day) from 12 to 16 weeks of age (n = 13). (**E**) Gene expression in the aorta after Ang II infusion in ApoE^*−/−*^ mice treated with vehicle and α-MSH (n = 6). (**F**) Representative pictures of the aorta and (**G**) AAA incidence and (**H**) diameter after Ang II infusion in MC4R^*TB/TB*^ mice treated intraperitoneally with vehicle and α-MSH (1.0 mg/kg/day) for 4 weeks (n = 6–7). Correlation between the plasma α-MSH concentration and *MC4R* gene expression levels in cultured monocytes isolated from the peripheral blood of (**I**) healthy controls (n = 50) and (**J**) patients with T2DM (n = 33). *P < 0.05, **P < 0.01, ***P < 0.001.

In macrophages obtained from LysM (+);MC4R^*TB/TB*^ mice, LPS-induced protein kinase A, IκB, and p65 phosphorylation were significantly attenuated by the α-MSH pretreatment (Fig. 6A, Supplementary Fig. 6A). However, this effect was not observed in macrophages from LysM (−);MC4R^*TB/TB*^ mice. In ApoE^−/−^ mice, intraperitoneal α-MSH treatment significantly reduced the incidence and diameter of Ang II-induced AAA (Fig. 6B–D), which was accompanied with reduced expression of pro-inflammatory genes in the aorta (Fig. 6E). In contrast, the incidence or diameter of Ang II-induced AAA in MC4R^*TB/TB*^ mice was not affected by α-MSH treatment (Fig. 6E–H), which was consistent with our ex vivo observation (Fig. 6A). These results demonstrate the possibility that α-MSH exerts its effects through the activation of MC4R signaling and suppression of NF-κB activity and its downstream pro-inflammatory genes expression in macrophages.

### Significant correlation of plasma α-MSH concentration in diabetic subjects with atherosclerosis and *MC4R* gene expression levels in monocytes

As we had already collected the monocytes and plasma samples from human subjects with and without type 2 diabetes mellitus (T2DM) in our previous study^26^, we also investigated the clinical significance of the *MC4R* gene expression in monocytes and plasma α-MSH concentration in humans (Table 1). We observed a significant negative correlation between the *MC4R* gene expression in monocytes and the plasma α-MSH concentration both in subjects without and with T2DM (Fig. 6I,J). Furthermore, among subjects with T2DM, the plasma α-MSH concentration was higher in those with carotid plaques than in those lacking it (Supplementary Fig. 6B) and correlated positively with brachial–ankle pulse-wave velocity (Supplementary Fig. 6C). These findings raise the potential that in diabetic subjects with atherosclerosis, plasma α-MSH concentration is elevated to compensate for MC4R signaling in monocytes.

**Table 1:** Characteristics of control subjects and patients with type 2 diabetes.

	Control (n = 50)	T2DM (n = 33)	P-value
Females (n)	22	17	0.65
Age (years)	63 [45–80]	67 [40–86]	0.39
Duration of diabetes (years)	0	10 [1–40]	
Smoking (n)	20	17	0.37
BMI (kg/m^2^)	22.4 [17.3–28.4]	25.0 [17.0–38.7]	< 0.05
SBP (mmHg)	117 ± 10	130 ± 19	< 0.001
DBP (mmHg)	73 ± 8	72 ± 12	0.62
FPG (mg/dL)	96 ± 8	158 ± 61	< 0.001
HbA1c (%)	5.6 [5.0–6.3]	8.4 [6.6–14.6]	< 0.001
IRI (µU/mL)	2.8 ± 1.4	7.3 ± 7.1	< 0.001
HOMA-IR	0.7 ± 0.4	3.0 ± 3.7	< 0.001
TG (mg/dL)	90 ± 53	158 ± 108	< 0.001
HDL-C (mg/dL)	62 ± 14	52 ± 19	< 0.05
LDL-C (mg/dL)	119 ± 27	132 ± 49	0.13
AST (U/L)	20 ± 5	26 ± 11	< 0.05
ALT (U/L)	19 ± 8	28 ± 18	< 0.01
γ-GTP (U/L)	25 ± 14	42 ± 33	< 0.01
eGFR (mL/min/1.73 m^2^)	71.4 ± 9.66	76.3 ± 18.3	0.11
hsCRP (mg/L)	1.7 [0.5–7.1]	6.1 [1.8–10.6]	< 0.001
ABI	N/A	1.09 [0.75–1.24]	
baPWV (cm/s)	N/A	1604 [1,166–3,261]	
Maximal IMT^20^	N/A	1.1 [0.5–2.5]	

The data are expressed as mean ± SEM or median and interquartile range. The data were compared using Student’s *t*-test or the Mann–Whitney U test.

*T2DM* type 2 diabetes, *BMI* body mass index, *SBP* systolic blood pressure, *DBP* diastolic blood pressure, *FPG* fasting blood glucose, *HbA1c* hemoglobin A1c, *IRI* immunoreactive insulin, *HOMA-IR* homeostasis model assessment of insulin resistance, *TG* triglycerides, *HDL-C* high-density lipoprotein cholesterol, *LDL-C* low-density lipoprotein cholesterol, *AST* aspartate transaminase, *ALT* alanine transaminase, *γ-GT* γ-glutamyl transferase, *eGFR* estimated glomerular filtration rate, *hsCRP* high-sensitivity C reactive protein, *ABI* ankle–brachial index, *baPWV* brachial–ankle pulse-wave velocity, *IMT* intima–media thickness, *N/A* not available.

Moreover, in subjects with thoracic aortic aneurysm and/or AAA (Table 2), macrophages-like cells obtained from lesions in aneurysm by laser capture microdissection showed lower *MC4R* gene expression than those obtained from non-lesions in aorta (Supplementary Fig. 7A,B).

**Table 2:** Characteristics of patients with aortic aneurysm.

n	19
Age, years	76 ± 2
Female (n)	6
Diameter of aneurysm^22^	56 ± 2
Body mass index	23 ± 1
Diabetes (n)	5
SBP (mmHg)	146 ± 9
DBP (mmHg)	89 ± 6
ABI	1.15 ± 0.02
baPWV (cm/s)	1714 ± 92
Type of aortic aneurysm (n)	
Thoracic	8
Abdominal	11
(Thoracoabdominal)	3
Shape of an aneurysm (n)	
Fusiform	15
Saccular	4

*baPWV* brachial–ankle pulse-wave velocity, *ABI* ankle–brachial index.

## Discussion

In this study, we demonstrated that loss of MC4R exacerbates vascular diseases, such as AAA and atherosclerosis by using *Mc4r*-deficient mice. Furthermore, we confirmed MC4R in macrophages attenuates AAA by suppressing NF-κB activity and subsequent vascular inflammation.

In this study, we first confirmed the occurrence of a leptin-dependent mechanism explaining the high AAA occurrence in WD-fed MC4R deficient mice with Ang II infusion. Locally applied leptin in periaortic area is known to augment the medial MMP-9 synthesis and aortic aneurysm size, implying that PVAT derived leptin may act as a factor promoting AAA development induced by obesity^4^, however, the exact mechanism remains unknown. We demonstrated that leptin exerts its effect through PI3K-dependent upregulation of the *Spp1* gene expression in VSMCs. When OPN receptors in VSMCs were neutralized with the anti-CD44 antibody, it significantly suppressed the incidence and size of Ang II-induced AAA, suggesting that leptin, elevated by MC4R deficiency, promotes vascular vulnerability via OPN induction in VSMCs. However, whether PVAT-derived or circulating leptin regulates this need further investigation. Consistent with our findings, a previous study reported a significant increase in OPN expression levels in the VSMCs and medial thickening in the OPN-Tg mice than in non-Tg mice^27^. Moreover, their zymographic analysis confirmed the increased matrix metalloproteinases-2 or -9 in the OPN-Tg aorta, leading to vascular vulnerability. It is also worth noting that metformin downregulates OPN expression in aorta and inhibits Ang II-induced AAA formation^28^. Further studies are needed to determine whether inhibiting OPN action, especially in hyperleptinemic subjects, can be promising strategy to prevent AAA formation.

Importantly, the protective role of MC4R signaling in vascular damage was highlighted when a significant difference in AAA size and survival rate was observed between *ob/ob*;MC4R^*TB/TB*^ and *ob/ob* mice. Additionally, we could successfully demonstrate that myeloid cell-specific rescue of the *MC4R* gene expression significantly suppressed the Ang II-induced AAA incidence in mice. To the best of our knowledge, this is the first report to suggest that MC4R is functionally expressed in monocytes and macrophages and possibly involved in protecting from AAA in vivo. While further investigation is still needed to validate, we could detect MC4R protein expression on vascular lesion-infiltrating macrophages in ApoE KO mice. In this study, we also confirmed the MC4R’s function in BM-derived macrophages by showing an increase in the intracellular cAMP upon α-MSH treatment, which leads to suppression of LPS-induced phosphorylation of the p65 and NF-kB activity. In 2014, MC4R was reported to be expressed in enteroendocrine L cells and regulated peptide YY and glucagon-like peptide 1 (GLP-1) release in mice^24^*.* Moreover, MC4R was localized in L cells and regulated GLP-1 and peptide YY secretion from ex vivo human intestine. Additionally, fasting peptide YY levels and oral glucose-induced GLP-1 secretion were reduced in humans carrying a total loss-of-function MC4R mutation^25^. Although, a study reported that MC4R expression in L cells is marginal and may not affect GLP-1 secretion in mice^29^, it does not rule out the possibility of MC4R being functionally expressed in monocytes and macrophages and contributing significantly to protect against AAA. Further research is required to confirm its detailed functional expression in macrophages and determine precise crosstalk between MC4R and NF-κB signaling pathways.

In addition to the finding of MC4R’s involvement in attenuating AAA in mice, we found that α-MSH treatment could also inhibit Ang II-induced AAA incidence, which was accompanied by the reduction in inflammatory-related-genes expressions in the aortic lesion. Importantly, these beneficial effects of α-MSH were observed in ApoE^−/−^ but not in MC4R^*TB/TB*^ mice. Although the possibility is low, this may be attributed to the α-MSH-MC4R signaling in central nervous system and further research is needed to confirm whether MC4R signaling particularly in macrophages is responsible for α-MSH's beneficial impact on cardiovascular phenotype. Moreover, α-MSH is known to exhibit anti-atherosclerotic effects by binding to MC1R in the endothelial cells or macrophages^30, 31^. However, here, exogenous α-MSH administration did not affect Ang II-induced AAA formation in MC4R^*TB/TB*^ mice, suggesting that α-MSH inhibited Ang II-induced AAA formation through MC4R.

There are several limitations in this study. First, since ligands other than OPN can also bind with CD44, we can’t deny the possibility that the results of anti-neutralizing antibody treatment against CD44 were affected by inhibition of other ligand’s binding with CD44. Second, since other pathways such as cell cycle regulation and cholesterol metabolism were ranked higher than NF-κB signaling in Fig. 5G, it is possible that these pathways contributed to inhibition of Ang II-induced AAA development in LysM (+);MC4R^*TB/TB*^ mice. Since It is reported that surplus cholesterol efflux from macrophage is antiatherogenic^32^, the link of cholesterol metabolism and MC4R in macrophage should be addressed in further research. Third, hydralazine is known to stimulate sympathetic nerve system in addition to antihypertensive effects. Therefore, it is still possible that this effect might affect the AAA incidence in hydralazine-treated MC4R^*TB/TB*^ mice. Forth, we only used male mice in this study, these observations should not be extrapolated to the females as they may vary with the sex of the animal. Lastly, the sole use of the tail-cuff method to monitor blood pressure can be considered as another drawback, as telemetry is usually recommended for measuring subtle BP changes or sympathetic tone. However, because neither slight BP changes nor sympathetic tone are the main parameter being examined in this research, we think tail-cuff measurement is sufficient for this investigation. Despite these limitations, our study demonstrates the functional relevance of MC4R in macrophages. The synthetic agonist of MC4R, setmelanotide, was recently approved by the U.S. Food and Drug Administration to be used for chronic weight management in genetic obesity including leptin receptor deficiency^19^. Therefore, to explore the clinical potential of MC4R, further research investigating its actions in peripheral tissues/cells such as vascular and macrophages is needed.

## Methods

Additional details about the methods are provided in Online Supplemental Materials.

### Animals and experimental protocol

WT (C57BL/6J) mice were purchased from CLEA Japan, Inc. (Tokyo, Japan) and *ob/ob* and ApoE KO mice were purchased from Charles River Laboratories Japan, Inc. (Kanagawa, Japan). The MC4R^*TB/TB*^ mice with the C57BL/6J background were gifted by Dr. Joel K. Elmquist (University of Texas Southwestern Medical Center, Dallas, TX, USA). The animals had free access to water and were fed a SD (CE-2; 343 kcal/100 g, 12.6% energy as fat; CLEA Japan, Inc.). In the WD feeding experiments, 8-weeks-old mice were given a WD for 9 weeks (WD, D12079B, 41% fat, 43% carbohydrate, and 17% protein content on an energy basis, Research Diets Inc., New Brunswick, NJ, USA). ApoE KO male mice were fed an SD throughout the experimental period. From 13 weeks onwards, their bodyweight and blood pressure (BP-98A; Softron, Tokyo, Japan) were measured every week. In the hydralazine administration experiment, hydralazine (20 mg/kg/day) was administered as drinking water for 4 weeks to MC4R^*TB/TB*^ mice, and adjusted the systolic blood pressure (sBP) to that of the MC4R^+*/*+^ mice. Following the completion of the experiment, several tissues were dissected under intraperitoneal pentobarbital anesthesia (30 mg/kg).

### Ang II-induced AAA model

In the experiment of Ang II administration to induce AAA, 13-weeks-old male mice were anesthetized (30-mg/kg intraperitoneal pentobarbital) and infused with 500 ng/kg/min Ang II (Sigma-Aldrich, St Louis, MO) for 28 days. Ang II was delivered subcutaneously using ALZET model 2006 osmotic minipumps (DURECT Corp., Cupertino, CA, USA). The pumps were placed into the subcutaneous space of pentobarbital-anesthetized mice through a small incision in the back of the neck and incision was closed with surgical glue. All incision sites healed rapidly without any medication. In the experiment of α-MSH (M4135, Sigma-Aldrich, 1 mg/kg/day) or anti-CD44 neutralizing antibody (BE0039, BioXCell, 200 μg/day) treatment, they were injected intraperitoneally into ApoE KO or MC4R^*TB/TB*^ mice for 4 weeks starting on the day the minipumps were implanted. Aneurysm was defined as a 50% increase in maximum diameter of abdominal aorta.

### Atherosclerotic lesion analysis

For atherosclerotic lesion analysis, the aortas were pinned to silicon dishes and stained with Oil Red O to detect lipids as previously described^33, 34^. The areas were quantified using ImageJ software, and the results were expressed as percentage of the total aorta area.

### Human studies

For the measurement of biochemical parameter and gene expression, we used the serum/plasma and monocyte samples of healthy (n = 50) and diabetic (n = 33) subjects obtained in our previous study^26^: the plasma α-MSH concentration was measured using ELISA (CUSABIO Technology LLC, USA), and the gene expression in monocytes was quantified by qPCR. The carotid intima–media thickness (IMT), brachial–ankle pulse-wave velocity (baPWV), and ankle–brachial index^35^ were measured and evaluated as previously described^26, 35^. For human aortic aneurysm studies, diseased aortic samples were obtained from patients with aortic aneurysm in the thoracic (n = 8) and abdominal (n = 11) legions, including the thoracoabdominal legions (n = 3). Control aortic samples (n = 3) were obtained from three patients with vascular diseases without aortic aneurysm. The obtained tissues were immediately fixed with formalin and subjected to laser capture microdissection as described in supplementary methods.

### Statistical analysis

Normally distributed data were expressed as mean ± standard error of the mean and compared using Student’s *t*-test or analysis of variance with associated Tukey’s multiple comparisons test. Alternatively, for non-normally distributed data, nonparametric statistical analysis was performed using the Mann–Whitney or Kruskal–Wallis tests with Dunn’s post hoc test. *P* values < 0.05 were considered statistically significant. The statistical analysis was conducted using GraphPad Prism 7 (GraphPad Software, Inc., CA, USA).

### Institutional review board statement

All experiments were conducted strictly in accordance with ARRIVE guidelines and the Guide for the Care and Use of Laboratory Animals of the Tokyo Medical and Dental University and the University of Yamanashi. The protocol was approved by animal research committees in Tokyo Medical and Dental University (#2013-025C4), University of Yamanashi (#1668 and #2286), and Isawa Hot Spring Hospital (#2018-003).

### Informed consent

Written informed consent was obtained from all participants before enrollment.

### Supplementary Information

Supplementary Information.

## Data Availability

The microarray datasets used in this article is available in the https://www.ncbi.nlm.nih.gov/geo/query/acc.cgi?acc=GSE198975.

## Abbreviations

MC4R: Melanocortin-4 receptor
AAA: Abdominal aortic aneurysm
OPN: Osteopontin
LysM: Lysozyme
NF-kB: Nuclear factor-kappa B
Ang II: Angiotensin II
α-MSH: α-Melanocyte-stimulating hormone
baPWV: Brachial-ankle pulse wave velocity
