# Supplementary material for: Melanocortin-4 receptor in macrophages attenuated angiotensin II-induced abdominal aortic aneurysm in mice
Source: Sci Rep. 2023 Nov 13;13:19768. doi: 10.1038/s41598-023-46831-4 (PMC10643430; doi:10.1038/s41598-023-46831-4)
Supplement: Supplementary file 1 — Supplementary Information. [file 41598_2023_46831_MOESM1_ESM.docx]

**Online Supplemental Material**

**Melanocortin-4 receptor in macrophages attenuated angiotensin II-induced abdominal aortic aneurysm** **in mice**

Kentaro Mori,^1*^ Hideyuki Okuma,^1^ Suguru Nakamura,^1^ Hiroyuki Uchinuma,^1^ Shigeaki Kaga,^2^ Hiroyuki Nakajima,^2^ Yoshihiro Ogawa,^3^ and Kyoichiro Tsuchiya^1*^

**SUPPLEMENTARY METHODS:** Page number 2

**SUPPLEMENTARY FIGURE AND LEGENDS:** Page number 7

**REFERENCES:** Page number 14

**SUPPLEMENTARY METHODS**

**NF-κB luciferase assays**

We measured the luciferase activity in primary bone marrow-derived macrophages transfected with adenovirus containing an NF-κB reporter construct (Vector Biolabs) using the Dual-Luciferase Reporter Assay System (Promega) as previously described (1). We transduced macrophages at MOI 500, 18–24 h prior to the experiments. The reporter activity was normalized by protein concentration or co-transfected *Renilla* luciferase as appropriate.

**Laser capture Microdissection**

The macrophages in the aortic tissues were microdissected as previously described (2). Human aortic samples were formalin-fixed and paraffin-embedded (FFPE), followed by hematoxylin and eosin staining. The H&E slides having 5 μm sections were loaded onto the MMI CellCut Laser Microdissection system (Molecular Machines & Industries, 50102). Using the MMI CellCut software, a complete slide scan was conducted using a CellScan toolbar at a magnification of 4× to ease the navigation. Using a computer mouse, region of interests were selected using a closed-shape manual drawing tool: clusters of mononuclear cells in AAA lesions or control tissues were targeted as infiltrated cells including macrophages. Then, we focused our laser at 350 μm and performed an automated cutting using a 60% laser power setting moving at a rate of 50 μm/s. To collect microdissected specimens, we used adhesive MMI transparent caps (MMI, 50202), and MMIs CapLift technology was used to gently lift and store specimens onto the adhesive caps. RNA was extracted using the RNeasy FFPE Kit (QIAGEN, CA, USA), and first-strand cDNA, synthesized using the SuperScript™ III First-Strand Synthesis System (Life Technologies/Invitrogen, CA, USA), was subsequently processed for real-time quantitative reverse transcription polymerase chain reaction (qRT-PCR).

**Biochemical assays**

Blood was obtained from the tail veins or cardiac puncture. The blood glucose was measured using a glucometer (OneTouch Verio^®^ IQ; LifeScan Japan Co., Ltd., Tokyo, Japan). The concentrations of serum total cholesterol, NEFA, and TG were determined in Oriental Yeast Co., LTD. The concentrations of insulin (Morinaga Institute of Biological Science, Inc., Kanagawa, Japan), leptin (FUJIFILM Wako Shibayagi Co., Gunma, Japan), α-MSH (Biocompare), angiotensin II (LSBio, Seattle, USA), and cAMP (Cayman, Ann Arbor, USA) were measured using ELISA Kit.

**Histological analysis**

The aorta were fixed with 4% paraformaldehyde and embedded in paraffin, and 4-µm-thick sections were prepared to be mounted on slides. The sections were stained with H&E. Oil Red O (Sigma-Aldrich) staining was performed to visualize neutral lipids present in the atherosclerotic lesions. The macrophages in the aorta were immunohistochemically detected using a rat monoclonal F4/80 antibody (MCA497GA, AbD Serotec, Kidlington, UK). The vascular smooth muscle cells (VSMCs) in the abdominal aortic aneurysm (AAA) were stained as immunohistochemically detected using an α-smooth muscle actin (α-SMA) antibody (ab5694, Abcam, Cambridge, UK) and OPN antibody (ab8448, Abcam, Cambridge, UK). Fluorescent immunostaining was performed with α-SMA antibody (ab5694, Abcam), OPN antibody (ab8448, Abcam), CD68 antibody (MA5-13324, Thermo Fisher Scientific), and MC4R antibody (BS-11417R, Bioss antibodies). Elastica van Gieson and Masson trichrome staining were also performed in the AAA and atherosclerotic lesions. Primary antibody binding was visualized using a DAB^+^ chromogen (Dako, Glostrup, Denmark). Positive areas were measured using the NIH ImageJ software. All microscopic images were acquired using the Keyence BZ-9000 microscope.

**qRT-PCR**

The total RNA of the aorta was isolated using the Sepasol reagent (Nacalai Tesque, Inc.). RNA was reverse-transcribed using Random Primer (Thermo Fisher Scientific Inc., Waltham, MA, USA) and ReverTra Ace (Toyobo Co., Ltd., Osaka, Japan). qRT-PCR was performed using the StepOnePlus Real-Time PCR System with Fast SYBR Green Master Mix Reagent (Thermo Fisher Scientific Inc.). The data were normalized to the 36b4 level and analyzed using the comparative CT method.

**Western blotting**

The tissues and cells were homogenized in a lysis buffer (2% SDS, 4 M urea, 1 mM EDTA, 150 mM NaCl, 50 mM Tris pH 8.0). Immunoblotting was performed with phospho (Ser32)-IκBα (2859, Cell Signaling Technology), total IκBα (9242, Cell Signaling Technology), phospho-NF-κB p65 (Ser536) (3033, Cell Signaling Technology), NF-κB p65 (3034, Cell Signaling Technology), phospho-PKA (Thr197) (5661, Cell Signaling Technology), PKA (4782, Cell Signaling Technology), β-actin (4970, Cell Signaling Technology), and MC4R (sc-55567, Santa Cruz) antibodies. Immunoblots were detected and analyzed using ECL Prime Western Blotting Detection Reagent and ImageQuant LAS 4000 mini (GE Healthcare, Little Chalfont, UK).

**Isolation and culture of VSMC and bone marrow macrophages**

Vascular smooth muscle cells (VSMCs) were isolated and cultured from 4-weeks-old WT male mice as previously described (3). Primary mouse macrophages were isolated from the BM of 8–10-weeks-old WT male mice, as previously described (4). BM was flushed out with 10 mL phosphate-buffered saline (PBS) through a 23-guage needle. The clumps were gently disaggregated using a needle-less syringe and passed through a 70-µm cell strainer. The cell suspension was centrifuged at 250 g at room temperature for 5 min to pellet the cells. The supernatant was discarded, and the cells were resuspended in Dulbecco’s Modified Eagle Medium (DMEM; Invitrogen, St. Louis, MO, USA) supplied with 10% fetal bovine serum (FBS) and 1% L-glutamine (Invitrogen). M-CSF was added at 25 ng/mL on days 0 and 3, and then, the following experiment started from day 5.

**Microarray analysis**

The microarray analysis was conducted using the Clariom™ S Assay, mouse (Thermo Fisher Scientific Inc., USA). The total RNA was extracted from the cultured BM-derived macrophages of a mouse. For the macrophage pathway analysis, genes that were increased by >1.5-fold in LysM-Cre(-);*MC4R^TB/TB^* mice in comparison to LysM-Cre(+);*MC4R^TB/TB^* mice were selected. The microarray data has been deposited in the NCBI Gene Expression Omnibus under an accession code.

**Monocyte isolation**

Monocyte isolation from human subjects was performed as previously described (5). Briefly, approximately 25 mL of the fasting anti-coagulated blood sample was diluted with an equal volume of lymphocyte separation solution (Nacalai Tesque, Japan). The samples were centrifuged at 400 g in a swing-out rotor for 30 min at 22 °C, and the peripheral blood mononuclear cell (PBMC) layers were harvested using a pipette. The PBMCs were repeatedly washed using DMEM (Thermo Fisher Scientific Inc., Waltham, MA, USA) supplemented with 1% penicillin/streptomycin (P/S) containing 10% FBS. The PBMCs were cultured in collagen-coated multi-well plates in DMEM with 1% P/S containing 10% FBS for 2 h in an incubator. The PBMCs were washed with PBS to remove the lymphocytes (6,7). Monocyte purity was verified by FACSCelesta (Becton, Dickinson and Company, NJ, USA) using phycoerythrin (PE)-conjugated anti-human CD14 antibody (BioLegend, CA, USA) (5). For serum starvation, the media was replaced with DMEM containing 0.25% bovine serum albumin and incubated for 2 h.

**List of primers**

|  | Fw | Rv |
| --- | --- | --- |
| *Tnf* | TCTTCTGCCTGCGCACTTT | GTCACTCGGGGTTCGAGAAG |
| *Ccl2* | ATCCCAATGAGTAGGCTGGA | GAGCTTGGTGACAAAACTACA |
| *Ccl5* | CTGCCCTCACCATCATCCTC | AGAGGTAGGCAAAGCAGCAG |
| *Emr1* | GTGGAGGCAGTGATGCTCTT | TGGAAGCCCATAGCCAAAGG |
| *Tgfb1* | CCTGAGTGGCTGTCTTTGA | GGGCTGATCCCGTTGATTTTC |
| *Vcam1* | CTGGGAAGCTGGAACGAAGT | GCCAAACACTTGACCGTGAC |
| *Il1b* | CTGGTGTGTGACGTTCCCATTA | CCGACAGCACGAGGCTTT |
| *Nos2* | CACTGCCCGGGAAATGTTTG | CATAGCGGATGAGCTGAGCA |
| *Cxcl2* | GGCGGTCAAAAAGTTTGCCT | TTCTTCCGTTGAGGGACAGC |
| *mMc4r* | GCTTCTGACCCTGCTCCTA | GTATACATGCCATGGTGGTG |
| *hMC4R* | GCACACTTCTCTGCACCTCT | TCGTAGCACCCTCCATCAGA |
| *Lep* | TCCAGGATGACACCAAACCC | TGAAGTCCAAGCCAGTGACC |
| *Spp1* | AAAGGGCAGCCATGAGTCAA | GACTGATCGGCACTCTCCTG |

**SUPPLEMENTARY FIGURE AND LEGENDS**

**Supplementary Figure 1. Incidence of Ang II-induced AAA in MC4R*^TB/TB^* mice with or without treatment of hydralazine**

(A) Bodyweight, (B) sBP, and (C) Quantification of elastin break number in the aorta in 14-weeks-old WD-fed MC4R*^+/+^* and MC4R*^TB/TB^* mice during infusion of Ang II (500 ng/kg/min) for 28 days (n = 11–15 at the beginning of Ang II infusion). (D) The changes in systolic blood pressure (sBP) in WD-fed MC4R*^+/+^* and MC4R*^TB/TB^* mice treated with or without hydralazine (Hydra, 20 mg/kg/day), concomitantly administered with Angiotensin II (500 ng/kg/min) from 14 to 18 weeks of age (n=8). (E) Incidence and (F) diameter of Ang II-induced AAA in WD-fed WT and MC4R*^TB/TB^* mice treated with or without hydra (n=8). Representative pictures of (G) F4/80 staining and (H) quantification of the F4/80-positive area in the aorta (n = 11–15). * *p* < 0.05, ** *p* < 0.01, *** p < 0.001.

**Supplementary Figure 2. Metabolic profile in MC4R*^+/+^*** **and MC4R*^TB/TB^* mice in an ApoE*^-/-^* background**

(A) Total cholesterol, (B) triglyceride, (C) NEFA, (D) glucose, and (E) serum insulin concentration in MC4R*^+/+^* and MC4R*^TB/TB^* mice in an ApoE*^-/-^* background at 28-weeks of age (n=7–8). * *p* < 0.05, ** *p* < 0.01, *** p < 0.001.

**Supplementary Figure 3. Metabolic profile in MC4R*^TB/TB^*, *ob/ob;*MC4R*^TB/TB^*, and *ob/ob* mice**

Changes in Serum (A) Total cholesterol, (B) triglyceride, (C) glucose, (D) insulin, and (E) NEFA concentration in WD-fed MC4R*^TB/TB^*, *ob/ob;*MC4R*^TB/TB^*, and *ob/ob* mice when fed ad-lib (n=5–9). * *p* < 0.05, ** *p* < 0.01, *** p < 0.001.

**Supplementary Figure 4. Plasma osteopontin concentration in MC4R^+/+^ and MC4R*^TB/TB^* mice**

(A) Plasma osteopontin (OPN) concentration in MC4R^+/+^ and MC4R*^TB/TB^* mice (n=6–7). (B) Correlation between plasma leptin and OPN concentration in MC4R*^+/+^* and MC4R*^TB/TB^* mice (n=6–7). (C) Representative pictures of immunostaining in the aorta without OPN primary antibody WD-fed MC4R*^+/+^* and MC4R*^TB/TB^* mice (scale bars represent 20 μm). * *p* < 0.05, ** *p* < 0.01, *** p < 0.001.

**Supplementary Figure 5. Reconstitution of *Mc4r* expression in myeloid cells of MC4R^TB/TB^ mice**

(A) Western blotting of MC4R of various tissues and cells in WT mice, including RAW 264.7 cells and mouse bone marrow (BM)-derived macrophages (Mφ). (B) Conditional re-expression is achieved through the insertion between the transcriptional start site and the *Mc4r* coding region of a transcriptional blocking (TB) cassette flanked with *loxP* sites (represented by triangles). Lysozyme M (LysM) Cre-dependent excision of the region between the *loxP* sites removes the TB cassette and re-establishes *Mc4r* expression in Cre-expressing myeloid cells only. (C) Systolic blood pressure (sBP) in Western diet (WD)-fed LysM-Cre (+);MC4R^TB/TB^ and LysM-Cre (-);MC4R^TB/TB^ during Angiotensin II (500ng/kg/min) infusion from 14- to 18-weeks of age (n=9).

**Supplementary Figure 6. Effect of α-MSH BM-derived macrophages from LysM-Cre (+);MC4R^TB/TB^ and MC4R^TB/TB^ mice and plasma α-MSH concentration of T2DM patients**

Quantification of protein kinase A, and IκBα phosphorylation in BM-derived macrophages from LysM-Cre (+);MC4R^TB/TB^ and MC4R^TB/TB^ mice (n = 2–3). (B) Plasma α-MSH concentration of T2DM patients with (n = 9) or without (n = 24) carotid plaque (≥1.1 mm). (C) Correlation between the plasma α-MSH concentration and baPWV in patients with T2DM (n = 33). * *p* < 0.05, ** *p* < 0.01, *** p < 0.001.

**Supplementary Figure 7. Laser capture microdissection of mononuclear cells from lesion of human aortic aneurysm**

(A) Representative pictures of a Hematoxylin and eosin staining section before (upper) and after (lower) laser capture microdissection. Clusters of mononuclear cells (dotted line) in lesion of human aortic aneurysm were targeted as infiltrated cells including macrophages. (B) MC4R gene expression levels in macrophages isolated from the non-lesions of the aorta (control, n = 3) and the lesions of thoracic and abdominal aortic aneurysm (TAA, n = 8 and AAA, n = 11, respectively) *via* laser capture microdissection.

**REFERENCES**

1. Tsuchiya K, Banks AS, Liang CP, et al. Homozygosity for an allele encoding deacetylated FoxO1 protects macrophages from cholesterol-induced inflammation without increasing apoptosis. Arterioscler Thromb Vasc Biol 2011;31:2920–2928.

2. Herrera JA, Mallikarjun V, Rosini S, et al. Laser capture microdissection coupled mass spectrometry (LCM-MS) for spatially resolved analysis of formalin-fixed and stained human lung tissues. Clin Proteomics 2020;17:24.

3. Adhikari N, Shekar KC, Staggs R, et al. International Society of Cardiovascular Translational. Guidelines for the isolation and characterization of murine vascular smooth muscle cells. A report from the International Society of Cardiovascular Translational Research. J Cardiovasc Transl Res 2015;8:158–163.

4. Weischenfeldt J, Porse B. Bone Marrow-Derived Macrophages (BMM): Isolation and Applications. CSH Protoc 2008;pdb prot5080.

5. Nakamura S, Mori K, Okuma H, et al. Age-associated decline of monocyte insulin sensitivity in diabetic and healthy individuals. Diab Vasc Dis Res 2021;18:1479164121989281.

6. Jumeau C, Awad F, Assrawi E, et al. Expression of SAA1, SAA2 and SAA4 genes in human primary monocytes and monocyte-derived macrophages. PLoS One 2019;14:e0217005.

7. Pegorier S, Stengel D, Durand H, Croset M, Ninio E. Oxidized phospholipid: POVPC binds to platelet-activating-factor receptor on human macrophages. Implications in atherosclerosis. Atherosclerosis 2006;188:433–443.
